# Polymicrobial Infection in an Immigrant Female at the United States-Mexico Border

**DOI:** 10.7759/cureus.51400

**Published:** 2023-12-31

**Authors:** Eshani Kishore, Frederick Gyabaah, Abhizith Deoker

**Affiliations:** 1 Internal Medicine, Texas Tech University Health Sciences Center El Paso, El Paso, USA

**Keywords:** migration, public health, border health, dengue fever, malaria

## Abstract

Malaria is a highly infectious disease transmitted through the bite of the Anopheles mosquito carrying the parasite of the Plasmodium genus; it presents with cyclical fevers, myalgias, and headaches. In the United States, the vast majority of malaria cases are reported in people who travel abroad, mainly to Africa.^ ^These cases are predominantly linked to Plasmodium falciparum or ovale and can be medically treated with artemisinin, chloroquine, or atovaquone-proguanil. We discuss a case of a 38-year-old female immigrant from Venezuela living at an immigration facility who presented to a hospital located on the United States-Mexico border with a two-day history of watery diarrhea, headache, and subjective fever. She had experienced mosquito bites and likely contracted the illness in Chiapas, Mexico during her trek from Peru to the United States. Her case was unique as she tested positive for dengue fever antibodies acquired from a previous infection and also contracted rhinovirus during her clinical course. Her diagnosis of malaria was confirmed with a peripheral blood smear that revealed ring forms with no gametocytes. This in tandem with her route of travel suggested infection with Plasmodium vivax. She was treated with chloroquine while the malaria culture was pending and continued to spike fevers every 24-36 hours while on medication. Once the culture was confirmed, she was treated with atovaquone-proguanil as maintenance therapy. She was subsequently discharged on primaquine for 14 days to prevent relapse.

## Introduction

Malaria is a parasitic infection caused by the Anopheles mosquito. The most common symptom of malaria is cyclic fevers that spike every two or three days and correspond to the life cycle of the Anopheles mosquito. Other possible symptoms include headache, malaise, myalgias, arthralgias, diarrhea, and cough. Malaria should be clinically suspected in any patient immigrating from an endemic area who presents with a constellation of the symptoms mentioned above [1]. The Anopheles mosquito transmits parasites of the genus Plasmodium, which causes malaria in endemic areas. The genus Plasmodium is an amoeboid intracellular parasite that accumulates malaria pigment (an insoluble metabolite of hemoglobin). The Plasmodium genus can prey on different vertebrates, on red blood cells, and in tissue. The five species that can infect humans are as follows: P. malariae, P. falciparum, P. vivax, P. ovale, and P. knowlesi, with falciparum being the most common [2].

The pathophysiology of malaria starts when sporozoites from the Anopheles mosquito’s saliva infect hepatocytes. “Sporozoite” is a term that refers to the primary and infectious stage of the mosquito. The sporozoites then attack hepatocytes and rapidly divide to form hundreds of schizonts. This life cycle stage leads to an increase in transaminases due to intrinsic liver damage. The schizonts rupture in under a month, dispersing merozoites into the bloodstream [3]. Anywhere from 2 x 10^3^ to 40 x 10^3^ merozoites can be released into the bloodstream. These merozoites will then invade red blood cells and cause hemolysis. Within the erythrocyte, the merozoites catabolize glucose into lactic acid, leading to lactic acidosis and hypoglycemia [4].

Patients with malaria usually present with symptoms a few weeks after the initial infection. These symptoms can include fever, cough, and chills. Additional potential symptoms include diarrhea, malaise, arthralgias, and myalgias. The classic presentation involves fever and shaking chills, followed by an even higher fever with diaphoresis. The periodicity noted for each organism is as follows: P. falciparum, P. ovale, and P. vivax with a fever that spikes every 48 hours (tertian fever) and P. malariae with a fever that spikes every 72 hours [5]. In clinical practice, most patients do not present with exact periodicity because groups of organisms are released into the bloodstream at various times following lysis of erythrocytes. This unpredictable dispersal corresponds to a spike in the fever pattern [6].

Malaria has a low prevalence in the United States. It is most common in Africa and certain countries in South and Southeast Asia, such as Bangladesh, Bhutan, India, Indonesia, Maldives, Myanmar, Nepal, Sri Lanka, and Thailand [7]. However, given the recent significant increase in the number of people immigrating to the United States from these regions, US physicians need to be able to identify and manage patients with malaria. This increase in migration is very evident on the US-Mexico border. An unprecedented number of asylum seekers and refugees with rare disease pathologies arrive at international crossings along the US-Mexico border, such as in El Paso, Texas [8].

We report the case of a 38-year-old female Venezuelan migrant living in an immigration facility in El Paso, Texas who presented to a hospital on the United States-Mexico border with symptoms of malaria. She received treatment with chloroquine, atovaquone-proguanil, and primaquine along with supportive care.

## Case presentation

A 38-year-old female immigrant from Venezuela with a past medical history of hypertension and migraine presented to the Emergency Department escorted by Border Patrol with complaints of nausea, non-bloody emesis, watery diarrhea, and headache for two days. The symptoms were initially reported when she was already detained in the immigration camp, and the patient was brought to the hospital for further evaluation. This patient had started her trip to the US from Peru two months prior. She had been in Panama and the Darién Gap about a month and a half prior, eventually reaching Mexico about two to three weeks before this hospitalization. She had been incarcerated for three days while on the south-Mexican border in Chiapas. She also mentioned that she had been exposed to mosquitoes in Panama and Mexico and noted that she arrived in the United States shortly after her incarceration in Mexico. Of note, her husband had contracted P. vivax malaria. He had been treated while in Mexico, two weeks after leaving Panama.

At the time of presentation to the emergency room, the triage provider reported that the patient triggered sepsis criteria and was subsequently admitted (Table 1). A review of vitals and labs revealed leukocytosis (14.9 x 10^3^ IU/liter), tachycardia (127 beats per minute), and low core body temperature (36.2 °C). However, she was breathing room air with normal blood pressure and respiratory rate.

**Table 1:** Patient's vital signs and laboratory values upon initial presentation

Test	Result	Reference values
White blood cell count	14.9 x 10^3^ IU/liter	4.5 x 10^3^ – 11 x 10^3^ IU/liter
Heart rate	127 beats per minute	60 – 100 beats per minute
Oral temperature	36.2 °C	36.4 – 37.6 °C
Hemoglobin	8.5 grams/deciliter	13.2 – 16.6 grams/deciliter
Hematocrit	24.9%	37 – 52%
Platelet count	27 x 10^3 ^platelets	15 x 10^3^ – 45 x 10^3 ^platelets
Prothrombin time	15.3 seconds	11 – 15 seconds
Blood urea nitrogen	16 milligrams/deciliter	6 – 24 milligrams/deciliter
Creatinine	0.8 milligrams/deciliter	0.74 – 1.35 milligrams/deciliter
Total bilirubin	3.2 milligrams/deciliter	0.2 – 1.2 milligrams/deciliter
Direct bilirubin	0.5 milligrams/deciliter	0.2 – 1.3 milligrams/deciliter
Serum lipase	910 IU/liter	0 – 160 IU/liter
Serum sodium	122 millimoles/liter	135 – 145 millimoles/liter
Serum potassium	3.3 millimoles/liter	3.6 – 5.2 millimoles/liter
Aspartate aminotransferase	59 IU/liter	8 – 48 IU/liter
Alanine aminotransferase	44 IU/liter	7 – 56 IU/liter

Initial laboratory studies revealed that the patient's hemoglobin and hematocrit were low at 8.5 grams/deciliter and 24.9% respectively. White blood cell count was elevated at 14.9 x 10^3^ IU/liter, and platelet count was low at 27 x 10^3 ^platelets/microliter. The platelets that were observed were large in appearance. Her prothrombin time (PT) was slightly elevated at 15.3 seconds but partial thromboplastin time (PTT) was within normal limits. Her blood urea nitrogen level (BUN) and creatinine were normal at 16 milligrams/deciliter and 0.8 milligrams/deciliter. There was concern for hemolysis due to elevated total bilirubin (3.2 milligrams/deciliter) and direct bilirubin levels (0.5 milligrams/deciliter). However, no schistocytes were present on peripheral blood smears. Her lipase was extremely elevated at 910 IU/liter, while her sodium was low at 122 millimoles/liter. She also had hypokalemia with initial values of 3.3 millimoles/liter.

Due to severe watery diarrhea, IV normal saline was administered with acetaminophen as needed. Malaria was heavily suspected due to her status as a migrant from an endemic region and her clinical presentation. However, to rule out other possible causes, a complete workup as per the institutional protocol was carried out, which included HIV, stool culture, blood culture, C. difficile, ova and parasite, H. pylori, and Hepatitis C. These labs all returned negative. However, due to the elevated lipase in her initial labs, an abdominal CT scan was administered to rule out acute pancreatitis. The CT findings were within normal limits with no evidence of pancreatic necrosis. Supportive care was provided through maintenance intravenous (IV) normal saline, Zofran for nausea, and acetaminophen for pain control. Her hyponatremia was successfully treated with an infusion of normal saline at 100 milliliters/hour, though she was asymptomatic with no weakness or dizziness before infusion. Her hypokalemia also resolved.

A peripheral blood smear was obtained and revealed ring forms within the erythrocytes, indicative of malaria. While her malaria PCR was pending confirmatory diagnosis, she was treated with chloroquine 1000 milligrams orally once daily for two days. This treatment decision was made because the drug is efficacious in the early stages of the disease and works by preventing the DNA replication of malaria [9]. She was then transitioned to atovaquone-proguanil 400-1000 milligrams/four tablets orally daily for three days after the PCR was found to be positive for Plasmodium vivax infection.

Results from the infectious disease workup revealed that she had elevated dengue fever antibodies. However, clinical suspicion for an acute dengue fever episode was low because she had not presented with any classical symptoms of dengue fever, such as abdominal pain, joint pain, and shortness of breath [10]. Moreover, her low platelet count was more likely due to her malaria rather than an acute case of dengue fever. Her platelet count began steadily improving with the replenishment of fluids, and her fever was treated symptomatically with acetaminophen. Rhinovirus was incidentally detected on respiratory BioFire, and its only clinical manifestation was reactive sublingual lymphadenopathy. Hence, she was treated supportively. She was eventually discharged on primaquine 15 milligrams twice per day orally for 12 days to treat the hypnozoite, or dormant, stage of malaria and prevent any future relapse.

## Discussion

Our patient was also co-infected with rhinovirus and had dengue fever antibodies. While research on the impact of dengue fever antibodies on malaria prognosis is scarce, one study has revealed that dengue has a protective effect in cases of dengue and malaria co-infection [11]. The study showed that dengue decreased the parasitic load and liver transaminase levels compared to malaria mono-infection [11]. The platelet count and hemoglobin levels in patients who were co-infected were higher than their peers who were mono-infected. This could explain why our patient responded as well as she did. Her liver transaminases were initially elevated upon admission; her aspartate aminotransferase (AST) was 59 IU/liter and her alanine aminotransferase (ALT) was 44 IU/liter at initial presentation. Upon discharge, her AST was 38 IU/liter and her ALT eventually dropped to 24 IU/liter. Her platelet count improved from 27 x 10^3^ platelets upon admission to 222 x 10^3^ platelets upon discharge, and she never required a platelet transfusion.

Unlike dengue fever, rhinovirus and malaria do not appear to be linked clinically in the sense that rhinovirus does not appear to play a protective role in malaria prognosis. Moreover, our patient was asymptomatic and did not have any characteristic symptoms such as rhinorrhea. However, malaria and rhinovirus use the same intercellular adhesion molecule (ICAM-1) molecule to multiply [12]. ICAM-1 facilitates cell-to-cell adhesion and antigen-specific immune responses through leukocyte movement [12]. In this case, however, there is scant evidence that the patient’s rhinovirus co-infection had a significant impact on her malaria course.

Treatments for malaria ought to be tailored based on the patient’s travel history and history of drug resistance or lack thereof in that area. The presence or absence of other comorbid conditions such as glucose-6-dehydrogenase deficiency should also be considered. The treatment is multifaceted. While chloroquine aims to decrease the number of parasites in the liver stage [13], primaquine targets the parasites in the dormant hypnozoite stage [13]. Atovaquone-proguanil diminishes the association of ubiquinone and the cytochrome b1c complex within the electron transport chain, which in turn disrupts the mitochondrial function of the parasites [13].

A wide range of treatments exist that help to reduce mortality and morbidity associated with malaria. The species of the infecting parasite, the density of parasitemia in the region, and the severity of symptoms are important factors to be evaluated when selecting a treatment regimen [14]. Chloroquine drugs, such as primaquine, remain the first-line treatment option for malaria. There are chloroquine-resistant regions, including certain parts of Mexico, Panama, and areas west of the Panama Canal [15]. Based on our patient's clinical presentation, the most likely organism responsible for her condition was Plasmodium vivax, which is characterized by a fever that spikes every two days. The proven efficacy of chloroquine was the reason why it was the first drug administered to her and because it is difficult to definitively say where she contracted the illness. It was administered for two days. Chloroquine prevents the degradation of hemoglobin within erythrocytes, which prevents the parasite from growing and allows for the accumulation of hematin, which is a toxic agent to Plasmodium and leads to death [16].

Once the peripheral smear confirmed malaria, our patient was transitioned to atovaquone-proguanil therapy for three days. Atovaquone-proguanil functions by inhibiting the electron transport chain within parasites, and proguanil works synergistically by acting as a biguanide and enhances the effect of atovaquone [17]. Specifically, proguanil decreases the concentration of atovaquone needed to inhibit the mitochondrial membrane potential [17], thereby causing the death of the parasite by inhibiting its primary method of energy production. Our patient was eventually transitioned to primaquine to be taken for 12 days after discharge. Primaquine is an important medication for malaria treatment and it targets the hypnozoite phase, which is the dormant and replicative stage of the disease. The rate of malaria relapse is high, approximately 20% in patients who do not take primaquine. Hence, the medication was provided to our patient upon discharge to prevent re-infection. The malaria PCR eventually came back after her discharge and confirmed infection with Plasmodium vivax.

Due to its geographic location on the border, healthcare workers in El Paso regularly interact with a diverse population presenting with disease pathologies that are otherwise relatively uncommon in the United States, such as malaria. Refugees coming to the United States endure difficult and unsafe journeys; they often reside in temporary shelters with minimal sanitation, which exposes them to various infections. Given a presentation of cyclic fevers and diarrhea, malaria should be on the differential for any patient with significant international travel history and/or previous residence in a malaria-endemic area. Treatment should not be delayed for confirmatory diagnosis through a peripheral blood smear. Patients infected with severe malaria can suffer from lifelong impairments such as loss of kidney function, eyesight, and eventually death [18].

## Conclusions

This case report described the clinical course of a 38-year-old female who had recently immigrated to the United States from Venezuela and presented with relapsing fevers, diarrhea, and headaches and was eventually diagnosed with malaria. Incidentally, she was also found to have rhinovirus and positive dengue fever antibodies. Her treatment course involved chloroquine with a transition to atovaquone-proguanil based on results from the peripheral blood smear that demonstrated ring forms and no gametocytes. Her pattern of fever spiking every two days and her past travel history made Plasmodium vivax the most likely causative organism. Confirmation of the suspected organism was later provided with malaria PCR after discharge. Our patient responded well to treatment and, despite having electrolyte abnormalities such as hyponatremia, hypokalemia, elevated transaminases, large platelets, and elevated lipase, her labs improved eventually. The normal saline, IV fluids, and a regular diet that were provided to her during her hospital course greatly improved the state of dehydration and malnourishment that she had initially demonstrated.
